# 晚期肺鳞癌治疗进展

**DOI:** 10.3779/j.issn.1009-3419.2020.101.35

**Published:** 2020-10-20

**Authors:** 鸣 高, 清 周

**Affiliations:** 1 510515 广州，南方医科大学第二临床医学院 The Second School of Clinical Medicine, Southern Medical University, Guangzhou 510515, China; 2 510080 广州，广东省医学科学院广东省人民医院，华南理工大学医学院，广东省肺癌研究所，广东省肺癌转化医学重点实验室 Guangdong Lung Cancer Institute, Guangdong Provincial Key Laboratory of Translational Medicine in Lung Cancer, Guangdong Provincial People's Hospital, Guangdong Academy of Medical Sciences, School of Medicine, South China University of Technology, Guangzhou 510080, China

**Keywords:** 肺肿瘤, 化疗, 靶向治疗, 免疫治疗, Lung neoplasms, Targeted therapy, Chemotherapy, Immunotherapy

## Abstract

肺癌是世界及中国发病率和死亡率第一的恶性肿瘤，肺鳞癌因其独特的临床表现、病理表现及基因突变特征，在诊治中与肺腺癌的区别显著，在临床研究中也作为单独的类型进行探索。除了肺鳞癌常表现为高龄、中央型肿瘤、诊断分期晚、合并症多，其往往少有驱动基因突变使得靶向治疗难以在肺鳞癌患者中获益，但是对于肺鳞癌的靶向治疗探索并未停止，包括纤维母细胞生长因子受体-酪氨酸激酶抑制剂和细胞周期蛋白依赖性激酶4和6抑制剂等新药在肺鳞癌患者中进行了多项I期研究。但随着免疫治疗的发展，鳞癌患者因其基因突变复杂，肿瘤突变负荷高等特点，免疫治疗联合化疗终于使肺鳞癌患者的预后有所改善。本文旨在从化疗、靶向治疗、抗血管治疗及免疫治疗几方面综述近年来晚期肺鳞癌的治疗进展。

## Abstract (en)

Lung cancer ranks first both in mobidity and motality in china and worldwild. Squamous cell lung carcinoma is distinguished from lung adenocarcinoma in diagnosis and treatment due to its unique clinical & pathological manifestations and gene mutation characteristics, as a result, it is also explored as a separate type in clinical researches. In addition to the clinical manifestations of elderly, central tumors, late staging at diagnosis, and multiple comorbidities, the lack of driver genes makes it nearly impossible for these patients to benefit from targeted therapy. However, the exploration of targeted therapy for lung squamous cell carcinoma has never stopped. Several new drugs including fibroblast growth factor receptor tyrosine kinase inhibitor and cyclin dependent kinase 4 and 6 inhibitors have been studied in multiple phase I studies specifically in patients with lung squamous cell carcinoma. However, with the development of immunotherapy, the characteristics such as complex gene mutation and high tumor mutation burden makes it possible for patients with squamous cell lung cancer to benefit from immunotherapy combined with chemotherapy. This review will provide an overview of the treatment progress in advanced squamous cell lung cancer including chemotherapy, targeted therapy and immunotherapy.

21世纪以来肺癌的治疗模式变化巨大，预后不断改善，2010年-2014年诊断的中国肺癌患者5年生存率已经可以达到20%-30%，世界范围内，肺癌患者的生存自2000年以来改善了5%-10%，而中国肺癌患者的生存改善甚至超过了10%^[1]^。60%以上的肺腺癌可以找到驱动基因^[2]^，包括人表皮生长因子受体（epidermal growth factor receptor, *EGFR*）、棘皮动物微管相关蛋白样4（echinoderm microtubule-associated protein-like 4, *EML4*）/间变性淋巴瘤激酶（anaplastic lymphoma kinase, *ALK*）和*c-ros*原癌基因1酪氨酸激酶（c-ros oncogene 1 receptor tyrosine kinase, *ROS1*）基因融合等，并且针对上述突变的靶向药物不断推陈出新，明显改善了肺腺癌患者的生存。但对于肺鳞癌，虽然占全部肺癌的25%-30%^[3]^，但常见驱动基因如*EGFR*突变和*ALK*基因重排的发生率很低，分别为约2.7%和1.5%-2.5%^[4-6]^，因此仅有少数鳞癌患者有机会接受EGFR-酪氨酸激酶抑制剂（EGFR-tyrosine kinase inhibitor, EGFR-TKI）或ALK抑制剂治疗。此外由于肺鳞癌独特的临床病理特点如中央型肿瘤、高龄、诊断时分期晚、合并症较多等，使其治疗充满挑战，但也正因为肺鳞癌基因突变复杂，反而成为可能从免疫治疗获益的人群。除了免疫治疗，还有其他机制及通路的积极探索。本文旨在从化疗、靶向治疗、抗血管治疗及免疫治疗几个方面回顾、总结近年来晚期肺鳞癌的治疗进展，展望未来探索趋势。

## 1 化疗

众所周知，绝大部分肺鳞癌无明确驱动基因，因此即使在TKI改变肺癌治疗模式的时代，化疗仍在晚期肺鳞癌的治疗中有着不可替代的地位。含铂双药方案是驱动基因阴性肺鳞癌的标准治疗，为了提高含铂方案的耐受性，日本和中国学者都在探索奈达铂作为新型铂类治疗晚期肺鳞癌。来自日本的奈达铂联合多西他赛对比顺铂联合多西他赛一线治疗晚期肺鳞癌的研究结果^[7]^显示奈达铂组较顺铂组的生存略有优势[中位总生存期（overall survival, OS）13.6个月*vs* 11.4个月，HR=0.81（95%CI: 0.65-1.02），*P*=0.037]，奈达铂组降低3级以上非血液学毒性发生率（包括恶心、乏力、低钠血症和低钾血症等）的同时增加了3级以上血液学毒性发生率（包括白细胞减少、中性粒细胞减少及血小板减少）。来自我国的JUST研究结果^[8]^显示意向性治疗人群中奈达铂联合多西他赛组的中位无疾病进展生存期（progression-free survival, PFS）与对照组无明显差异：PFS 4.63个月（95%CI: 4.43-5.10）*vs* 4.23个月（95%CI: 3.37-4.53）[HR=0.778 (90%CI: 0.599-1.009), *P*=0.056, 4]，奈达铂组降低了3级以上非血液学毒性（包括恶心呕吐和肌酐异常）和部分血液学毒性（贫血）反应的发生率，但其3级以上血小板减少发生率高于对照组。因此对于不能耐受顺铂或卡铂的患者，奈达铂可作为晚期肺鳞癌患者一线治疗的另一个选择。除了铂类以外的化疗药物也在进行探索，白蛋白紫杉醇联合卡铂对比紫杉醇联合卡铂一线治疗的Ⅲ期研究鳞癌亚组中^[9]^，白蛋白紫杉醇组的客观缓解率（objective response rate, ORR）高于对照组[41% *vs* 24%，缓解比值比（odds ratio, OR）=1.680（95%CI: 1.271-2.221），*P* < 0.001]，该方案也可以作为肺鳞癌晚期一线方案。

维持治疗是晚期肺腺癌诱导治疗后的标准治疗模式。晚期肺鳞癌的同药维持的IFCT-GFPC 0502研究^[10]^显示，吉西他滨联合顺铂诱导化疗后单药吉西他滨维持治疗组较观察组显著延长了PFS[3.8个月*vs* 1.9个月，HR=0.56（95%CI: 0.44-0.72），*P* < 0.001]。另一项Ⅲ期临床试验结果^[11]^也显示：一线吉西他滨联合顺铂诱导化疗后单药吉西他滨维持治疗组对比最佳支持治疗组显著延长了至疾病进展时间（time to progression, TTP）（3.6个月*vs* 2.0个月，*P* < 0.001），在基线行为状态评分（Karnofsky performance status, KPS） > 80分的患者有明显的总生存获益[中位OS 25.3个月*vs* 12.2个月，HR=2.1（95%CI: 1.2-3.8）]。一项换药维持的Ⅲ期临床研究结果^[12]^显示：一线吉西他滨联合顺铂化疗后即给予多西他赛维持治疗组较疾病进展后二线给予多西他赛治疗组PFS显著延长（5.7个月*vs* 2.7个月，*P*=0.000, 1），中位OS也有获益趋势（12.3个月*vs* 9.7个月，*P*=0.085, 3）。因此一线吉西他滨联合铂类诱导化疗后疾病控制有效且KPS评分较好的患者，可行吉西他滨单药维持治疗，换药维持的选择包括多西他赛。

特殊患者如体能状态（performance status, PS）2分及老年患者的治疗需要慎重权衡获益与毒性后进行选择。一项针对PS 2分的晚期非小细胞肺癌（non-small cell lung cancer, NSCLC）的Ⅲ期临床研究^[13]^对比了紫杉醇联合卡铂方案与吉西他滨或长春瑞滨单药治疗的疗效与安全性。虽然双药联合组的TTP较单药组有优势（4.6个月*vs* 3.5个月，*P* < 0.001），但中位OS并无统计学差异（8.0个月*vs* 6.6个月，*P*=0.184），同时双药联合组的3级-4级毒性反应发生率高于单药组（40% *vs* 22%）。一项来自意大利MILES研究^[14]^对比了吉西他滨联合长春瑞滨和吉西他滨或长春瑞滨单药在老年（≥70岁）NSCLC患者中的疗效，结果显示：联合化疗组较单药组无生存获益。然而对比了紫杉醇联合卡铂（紫杉醇每周给药，卡铂每月给药）和吉西他滨或长春瑞滨单药治疗老年NSCLC的Ⅲ期研究（IFCT-0501研究）结果却不同^[15]^：联合化疗组较单药组取得了明显的中位OS获益[10.3个月*vs* 6.2个月，HR=0.64（95%CI: 0.52-0.78），*P* < 0.000, 1]。同时联合化疗组3级-4级血液学毒性、乏力和感觉神经毒性也相应增加。上述研究提示对于PS 2分及老年患者，联合化疗并非禁忌，需要充分评估患者状态、器官功能及伴随疾病等个体化制定化疗方案。

二线治疗的方案为多西他赛单药或吉西他滨单药化疗。2000年发表的一项针对一线含铂方案化疗后疾病进展的晚期NSCLC患者的Ⅲ期随机临床研究^[16]^结果显示虽然多西他赛组的有效率高于长春瑞滨和异环磷酰胺[10.8% *vs* 6.7% *vs* 0.8%，多西他赛（docetaxel, D）100 mg/m^2^ *vs*长春瑞滨或异环磷酰胺（vinorelbine or ifosfamide, V/I），*P*=0.001；D 75 mg/m^2^ *vs* V/I *P*=0.036；D *vs* V/I，*P*=0.002]，但三组的总生存未见统计学差异。2017年发表的日本一项S-1对比多西他赛治疗含铂化疗失败的NSCLC的Ⅲ期研究中（东亚S-1研究）入组了60.1%-62.7%的日本患者^[17]^，33.4%-33.7%的中国患者，结果显示S-1对比多西他赛显示出非劣效性，中位OS分别为12.75个月*vs* 12.52个月（HR=0.945, 95%CI: 0.833-1.073, *P*=0.381, 8）。中位PFS也无明显差异（HR=1.033, 95%CI: 0.913-1.168），ORR分别为8.3% *vs* 9.9%。S-1组的不良反应以消化道为主，多西他赛不良反应以血液学为主。研究中鳞癌患者占17%-18.2%，在鳞癌亚组OS的HR为0.883（95%CI: 0.657-1.186），相比于腺癌患者HR=1.008（0.870-1.167），似乎更能从S-1的治疗中获益，而且S-1作为口服药物，相比于静脉给药更具便利性。综上，晚期肺鳞癌的二线化疗选择除了多西他赛、吉西他滨，还包括长春瑞滨、异环磷酰胺及S-1。

化疗仍是晚期肺鳞癌治疗的基石，一线除了传统的标准含铂双药化疗之外，还有奈达铂可以作为不耐受顺铂/卡铂患者的新选择，白蛋白紫杉醇较紫杉醇在联合卡铂一线治疗晚期肺鳞癌时显示出优效性；对于体力状态好的患者，诱导化疗后吉西他滨维持能进一步改善生存；对于体力评分差的患者，单药化疗是标准治疗，联合化疗要结合具体情况权衡毒性与获益；对于老年患者，联合方案的疗效获益于毒性伴行，需全面评估一般状况、器官功能及伴随疾病等因素。晚期肺鳞癌的二线治疗可选方案除了多西他赛或吉西他滨单药，还有长春瑞滨、异环磷酰胺，S-1也在东亚患者验证了二线相比于多西他赛的非劣效性，可以作为不耐受其他治疗的替代选择。

## 2 靶向治疗

美国国立综合癌症网络（National Comprehensive Cancer Network, NCCN）指南推荐不吸烟或较少吸烟患者常规进行*EGFR*和*ALK*检测，若有基因突变应首先应用相应的TKI治疗常见。但驱动基因如*EGFR*突变和*ALK*基因重排在肺鳞癌中发生率很低，因此有机会接受EGFR或ALK抑制剂治疗的患者仅占少数。

一项国际、多中心、随机、Ⅲ期临床研究^[18]^（TITAN研究）对比了厄洛替尼和多西他赛二线治疗含铂双药化疗后疾病进展且不经基因状态筛选的NSCLC患者，其结果显示出非劣效性[中位OS 5.3个月*vs* 5.5个月，HR=0.96（95%CI: 0.78-1.19），*P*=0.73]。LUX-LUNG8研究^[19]^对阿法替尼与厄洛替尼作为晚期肺鳞癌患者二线治疗的疗效进行了比较。与厄洛替尼相比，阿法替尼取得了更显著的PFS优势[中位PFS 2.6个月*vs* 1.9个月，HR=0.81（95%CI: 0.69-0.96），*P*=0.010, 3]和总生存获益[中位OS 7.9个月*vs* 6.8个月，HR=0.81（95%CI: 0.69-0.95），*P*=0.007, 7]。在LUX-LUNG8第二次分析中发现二代测序行肿瘤基因学检测的245例患者的亚组中^[20]^，53例（21.6%）的患者有*ERBB*突变阳性，这部分突变阳性的患者更能从阿法替尼治疗中获益[中位PFS 4.9个月*vs* 3.0个月，HR=0.62（95%CI: 0.37-1.02），*P*=0.06；mOS 10.6个月*vs* 8.1个月，HR=0.75（95%CI: 0.47-1.17），*P*=0.21]。EGFR-TKI在晚期肺鳞癌二线治疗的成功探索为不能接受化疗药物的患者提供了选择。

除了EGFR-TKI用于晚期肺鳞癌，抗EGFR的单克隆抗体Necitumumab也在晚期肺鳞癌进行了探索。在一项对比Necitumumab联合吉西他滨/顺铂和单纯吉西他滨联合顺铂化疗用于晚期肺鳞癌一线治疗的Ⅲ期临床研究^[21]^中，Necitumumab联合化疗组取得生存获益[中位OS 11.5个月*vs* 9.9个月，HR=0.84（95%CI: 0.74-0.96），*P*=0.01]。该药物未在中国上市，但为抗EGFR治疗在晚期肺鳞癌一线的作用提供了循证医学证据。

虽然肺鳞癌较少有*EGFR*突变和*ALK*重排，但肺鳞癌较肺腺癌的*EGFR*基因拷贝数增加及蛋白过表达等更为常见（82% *vs* 44%, *P* < 0.001）^[22]^，且鳞癌的总突变率很高并有显著的基因学复杂性^[23]^，包括纤维母细胞生长因子受体（fibroblast growth factor receptor, *FGFR*）基因突变、磷脂酰肌醇3激酶基因（phosphatidyl inositol 3 kinase gene, *PI3K*）突变、*EGFR*、磷酸酶和凝集素同源基因（phosphate and tension homology deleted on chromosome ten, *PTEN*）等，但遗憾的是针对上述靶点的TKI目前为止均未能取得成功并改变临床实践。

FGFR的抑制剂AZD4547治疗FGFR阳性经治晚期肺鳞癌的Ⅱ期研究因为缺乏疗效而终止^[24]^。其他正在进行临床研究的FGFR抑制剂包括Erdafitinib和Rogaratinib。2019年*Lancet Oncology*杂志发表了口服泛FGFR抑制剂Rogaratinib用于FGFR mRNA表达实体瘤的Ⅰ期研究^[25]^，期中NSCLC队列有20例患者，总体ORR为15%（95%CI: 8.6-23.5），耐受性尚可。目前已开放Rogaratinib在晚期经治FGFR mRNA过表达肺鳞癌患者的多中心单臂Ⅱ期研究。Erdafitinib和在标准治疗失败后*FGFR*基因突变的晚期肺鳞癌患者的Ⅱ期研究正在进行中。

针对PI3K通路的泛PI3K抑制剂Burparlisib和Taselisib均未能在PI3K通路活化的经治晚期肺鳞癌患者的Ⅰ期研究达到主要终点^[26, 27]^。针对其他基因如盘状结构域受体激酶2（discoidin domain receptor 2, DDR2）抑制剂也因毒性太大、无效而终止临床研究^[28]^。甚至有细胞周期蛋白依赖性激酶4/6（cyclin dependent kinase 4 and 6, CDK4/6）抑制剂的探索，如Palbociclib也在细胞周期基因突变阳性的晚期经治鳞癌患者的Ⅰ期研究中ORR仅有6%（95%CI: 1%-15%）^[29]^，未能进一步研究；英国研究者进行了Vistusertib（哺乳动物雷帕霉素靶蛋白1和2的双抑制剂）联合紫杉醇用于晚期肺鳞癌的Ⅰ期研究^[30]^，该联合方案在鳞癌患者的耐受欠佳，ORR达到35%，中位PFS为5.8个月（95%CI: 2.76-21.25），但该方案的Ⅱ期研究因缺乏疗效已经终止。2017年欧洲临床肿瘤协会（European Society for Medical Oncology, ESMO）学术年会报道了一种下调热休克蛋白27（heat shock prote in 27, Hsp27）的药物Apatorsen（OGX-427）用于肺鳞癌的Ⅱ期研究^[31]^。因Hsp27参与凋亡逃逸，且在70%-98%的鳞状细胞癌中高度表达，而化疗诱导的Hsp27表达可能是一种早期耐药的机制，因此研究设计为吉西他滨/卡铂联合OGX对比吉西他滨/卡铂治疗Ⅲb期或IV期初治或复发肺鳞癌，但该研究未达到主要终点，两组PFS无统计学差异[148 d *vs* 196 d, HR=0.83 (95%CI: 0.53-1.29), *P*=0.35]。

虽然多种新型靶向药物的尝试未获成功，但在鳞癌常见突变靶点FGFR的继续研究和开拓常规通路和靶点以外的靶点及作用机制仍有重要意义。目前国际上晚期肺鳞癌可在二线应用的靶向治疗药物仍然以抗EGFR为主，如厄洛替尼、阿法替尼及Necitumumab。

## 3 抗血管治疗

虽然贝伐单抗等抗血管生成的靶向药物因不良反应考量避免用于肺鳞癌，但针对血管内皮生长因子受体2（vascular endothelial growth factor, VEGFR2）的单克隆抗体Ramucirumab在晚期肺鳞癌二线治疗的大胆探索却取得了可喜的进展。在一项随机Ⅲ期对比Ramucirumab联合多西他赛和单药多西他赛二线治疗晚期NSCLC患者疗效的临床研究中^[32]^，Ramucirumab联合多西他赛组较单纯化疗组显著延长了中位OS[10.5个月*vs* 9.1个月，HR=0.86（95%CI: 0.75-0.98），*P*=0.023]，鳞癌患者占比25%-27%，在鳞癌亚组OS获益趋势一致，为[9.5个月*vs* 8.2个月，HR=0.883（95%CI: 0.692-1.127），*P*=0.319]。抗血管TKI也在肺癌的二线治疗中有探索，Nintedatinib联合多西他赛对比多西他赛治疗经治晚期NSCLC的Ⅲ期LUME-Lung1研究中^[33]^，Nintedatinib联合组的PFS较对照组有统计学优势[3.4个月*vs* 2.7个月，HR=0.79（95%CI: 0.68-0.92），*P*=0.001, 9]，研究中鳞癌占比42%，在鳞癌亚组中，PFS获益与总体人群一致[HR=0.77 (95%CI: 0.62-0.96), *P*=0.02]。随后进行了Nintedatinib联合吉西他滨一线用于晚期肺鳞癌的Ⅰ期/Ⅱ期研究，安全性可控，ORR为31.3%^[34]^，然而尚未在肺鳞癌继续Ⅲ期临床研究。

中国自主创新的抗血管TKI安罗替尼有用于晚期NSCLC三线及后线治疗的Ⅱ期及Ⅲ期临床研究中有纳入非中央型/空洞性肺鳞癌^[35, 36]^，虽然比例仅有10%-23%，Ⅲ期研究的鳞癌亚组（*N*=101）观察到PFS的改善[HR=0.37（95%CI: 0.22-0.60）]，OS的获益趋势与总体人群一致[HR=0.73 (95%CI: 0.45-1.18), *P*=0.19]，因此中国临床肿瘤学会（Chinese Society of Clinical Oncology, CSCO）指南也将安罗替尼纳入到晚期驱动基因阴性肺鳞癌指南中，用于外周型鳞癌的三线治疗。

虽然出于血管相关不良反应考虑，抗血管治疗在肺鳞癌的应用曾经收到限制，但循证医学证据还是证明Ramucirumab可以安全应用于晚期肺鳞癌二线治疗并改善生存，晚期外周型无空洞的肺鳞癌的三线可使用安罗替尼治疗。

## 4 免疫治疗

近年来，免疫治疗在恶性肿瘤治疗中的进展迅速，甚至改变了多种实体瘤的标准治疗，肺癌正是其中之一。肺癌的免疫治疗主要指抗程序性死亡因子1（programmed death 1, PD-1）/程序性死亡因子-配体1（programmed death-ligand 1, PD-L1）和抗细胞毒T细胞淋巴细胞抗原4（cytotoxic T-cell lymphocyte antigen-4, CTLA-4）。主要的药物包括Nivolumab、Pembrolizumab、Atezolizumab、Durvalumab及Ipilimumab等。免疫治疗在肺鳞癌的临床研究总结见表 1。

**1:** 晚期肺鳞癌免疫治疗的Ⅱ/Ⅲ期临床研究 Phase Ⅱ/Ⅲ clinical trials of immunotherapy for advanced lung squamous cell carcinoma

Clinical trial	Phase	Patients with SqCLC (%)	Screening criteria of PD-L1 expression	Study design	Control group [mOS (95%CI) (mon)]	Experimental group [mOS (95%CI) (mon)]	HR (95%CI; P)
Second-line treatment for advanced SqCLC							
CheckMate 017^[38]^	Ⅲ	100	-	Nivolumab *vs* Docetaxel	6.0 (5.1-7.3)	9.2 (7.3-12.6)	0.59 (0.44-0.79, *P* < 0.001)
CheckMate 078^[39]^	Ⅲ	39-40	-	Nivolumab *vs* Docetaxel	7.9 (5.9-11.4)	12.3 (9.5-14.6)	0.61 (0.42-0.89)
KEYNOTE-010^[40]^	Ⅱ/Ⅲ	≈24	-	Pembrolizumab *vs* Docetaxel	-	-	0.74 (0.50-1.09)
POPLAR^[42]^	Ⅱ	34	-	Atezolizumab *vs* Docetaxel	8.6	10.1	0.80 (0.49-1.30)
OAK^[41]^	Ⅲ	26	-	Atezolizumab *vs* Docetaxel	7.7 (6.3-8.9)	8.9 (7.4-12.8)	0.73 (0.54-0.98, *P*=0.038, 3)
JAVELIN Lung 200^[37]^	Ⅲ	30-35	-	Avelumab *vs* Docetaxel	7.5 (5.9-10.6)	10.6 (8.8-18.5)	0.70 (0.48-1.01)
First-line monotherapy for advanced SqCLC							
KEYNOTE-042^[44]^	Ⅲ	36-39	TPS≥1%	Pembrolizumab *vs* chemotherapy	-	-	0.75 (0.60-0.93)
KEYNOTE-024^[43]^	Ⅲ	17-18	TPS≥50%	Pembrolizumab *vs* chemotherapy	-	-	0.35 (0.17-0.71)
CheckMate 026^[45]^	Ⅲ	24	≥1%	Nivoluamb *vs* chemotherapy	10.2	10.5	0.82 (0.54-1.24)
MYSTIC^[46, 47]^	Ⅲ	32	TC ≥25%	Durvalumab/Durvalumab+Tre-melizumab *vs* chemotherapy	-	-	0.89 (0.57-1.37)
First-line combination therapy for advanced SqCLC							
KEYNOTE-407^[48]^	Ⅲ	100	-	Carboplatin+Paclitaxel/nab-Paclitaxel±Pembrolizumab	11.6 (10.1-13.7)	17.1 (14.4-19.9)	0.71 (0.58-0.88)
IMpower131^[49]^	Ⅲ	100	-	Carboplatin+nab-Paclitaxel±Atezolizumab	ITT (*n*=340) 13.5 (12.2-15.1) TC3/IC3 *n*=44 10.2 (7.1-17.5)	ITT (*n*=343) 14.2 (12.3-16.8) TC3/IC3 *n*=47 23.4 (17.8-NE)	0.88 (0.73-1.05, *P*=0.158, 1) 0.48 (0.29-0.81)
CheckMate 227^[51]^	Ⅲ	28-30	-	Nivoluamb+Ipilimumab *vs* chemotherapy	9.2	15.0	0.62
^-^: not reported; mon: months; SqCLC: squamous cell lung cancer; PD-L1: programmed death-ligand 1; mOS: median overall survival; TPS: tumor proportion score; TC: tumor cells; IC: tumor-infiltrating immune cells; ITT: intention-to-treat; NE: not estimable.							

免疫治疗在肺癌的探索从晚期二线开始，Nivolumab、Pembrolizumab、Atezolizumab、Durvalumab和Avelumab等PD-1/PD-L1抑制剂均选择了多西他赛单药作为对照组进行Ⅲ期临床研究。除了Avelumab之外，其他几个免疫检查点抑制剂均验证了较多西他赛的优效性^[37-39]^。CheckMate 017和CheckMate 078分别在全球和中国人群证实了Nivolumab在晚期肺鳞癌二线治疗中较多西他赛的优效性，CheckMate 017研究中Nivolumab较对照组的中位OS改善3.2个月[中位OS 9.2个月*vs* 6.0个月，HR=0.59（95%CI: 0.44-0.79），*P* < 0.001]，CheckMate 078研究中中国患者的生存获益类似[中位OS 12.0个月*vs* 9.6个月，HR=0.68（95%CI: 0.52-0.90），*P*=0.000, 6]，提高了客观缓解率（16.6% *vs* 4.2%, *P*=0.000, 1），同时明显降低3级以上不良反应（10% *vs* 47%），且免疫治疗不良反应以皮疹、乏力为主。其中鳞癌亚组中，Nivolumab组和多西他赛组总生存期分别为12.3个月和7.9个月（HR=0.61）。KEYNOTE-010研究^[40]^发现，晚期肺鳞癌的二线治疗中，PD-1抑制剂Pembrolizumab 10 mg/kg组和2 mg/kg组均较多西他赛组显著延长了总生存期[10 mg/kg：12.7个月*vs* 8.5个月，HR=0.61（95%CI: 0.49-0.75），*P* < 0.000, 1；2 mg/kg：10.4个月*vs* 8.5个月，HR=0.71（95%CI: 0.58-0.88），*P*=0.000, 8]，同时3级-5级不良反应发生率也明显低于对照组（16% *vs* 13% *vs* 35%）。此外基于两项国际随机开放多中心临床研究——OAK研究^[41]^和POPLAR研究^[42]^，PD-L1抑制剂Atezolizumab也获批用于肺鳞癌的二线治疗。上述研究均对比了多西他赛和Atezolizumab单药在晚期NSCLC的二线治疗中的疗效。在OAK研究中，Atezolizumab组较多西他赛组取得了4.2个月的显著生存获益[13.8个月*vs* 9.6个月，HR=0.74（95%CI: 0.63-0.87），*P*=0.000, 4]，其中鳞癌亚组中Atezolizumab总生存也优于多西他赛，中位OS 8.9个月*vs* 7.7个月[HR=0.73 (95%CI: 0.54-0.98), *P*=0.038]。

在晚期肺鳞癌的一线治疗探索中，KEYNOTE-024、KEYNOTE-042、CheckMate 026和MYSTIC研究均在经过免疫标志物筛查的人群中采用了单药对比化疗的研究设计，区别在于PD-L1表达筛选标准不同。KEYNOTE-024研究^[43]^中，对于PD-L1表达比例*≥*50%的晚期NSCLC初治患者，单药Pembrolizumab组较含铂双药化疗组显著提高有效率（44.8% *vs* 27.8%），明显改善PFS[10.3个月*vs* 6.0个月，HR=0.50（95%CI: 0.37-0.68），*P* < 0.001]，其中，鳞癌亚组的PFS改善尤为显著（HR=0.35, 95%CI: 0.17-0.71）。再者，免疫治疗组比化疗组的6个月总生存率更高[80.2% *vs* 72.4%, HR=0.60 (95%CI: 0.41-0.89), *P* < 0.005]，3级-5级治疗相关不良反应（treatment-related adverse events, TRAE）发生率明显低于化疗组（26.6% *vs* 53.3%），Pembrolizumab组最常见的3级-5级不良反应为皮肤毒性、肺炎及结肠炎。后续进行的KEYNOTE-042研究^[44]^，入组标准将PD-L1表达比例降低到≥1%，仍然取得了阳性结果。研究中根据肿瘤细胞阳性比例分数（tumor proportion score, TPS）分成不同亚组，在各个亚组中，Pembrolizumab均较标准化疗有总生存的优势（TPS≥50%: HR=0.69, 95%CI: 0.56-0.85, *P*=0.000, 3; TPS≥20%: HR=0.77, 95%CI: 0.64-0.92, *P*=0.002, 0; TPS≥1%: HR=0.81, 95%CI: 0.71-0.93, *P*=0.001, 8）。在TPS≥1%的鳞癌亚组中HR=0.75（0.60-0.93），与总体人群获益趋势一致。Checkmate 026和MYSTIC研究分别是Nivolumab和Durvalumab在晚期肺癌一线治疗的Ⅲ期研究^[45-47]^。这两项研究均未取得阳性结果，但均观察到免疫治疗组的总生存获益趋势，这两项研究的失败不能全盘否定免疫治疗在晚期肺鳞癌一线的作用，可能在免疫标志物选择和筛选标准以及方案选择上仍有优化的空间。但也提示免疫治疗单药在用于晚期肺鳞癌的一线治疗可能力度有限，因此也有了免疫联合方案在一线的探索。

KEYNOTE-407和IMpower131是近期报告的两项针对鳞癌患者的晚期一线Ⅲ期临床研究^[48, 49]^，均在未经标志物选择的晚期肺鳞癌一线患者对比免疫治疗联合化疗和标准化疗。KEYNOTE-407研究中，卡铂+紫杉醇/白蛋白紫杉醇联合Pembrolizumab较单纯化疗明显改善了生存，中位OS 15.9个月*vs* 11.3个月（HR=0.64, 95%CI: 0.49-0.85, *P* < 0.001）。中位PFS 6.4个月*vs* 4.8个月（HR=0.56, 95%CI: 0.45-0.70, *P* < 0.001)。两组不良反应发生率类似，联合组69.8% *vs*对照组68.2%，联合组因不良反应停药的比例更高（13.3% *vs* 6.4%）。IMpower131研究则获得了总体阴性的研究结果。Atezolizumab联合卡铂/白蛋白紫杉醇对比卡铂/白蛋白紫杉醇的OS未达到统计学差异，ITT人群的中位OS为14.2个月（95%CI: 12.3-16.8）*vs* 13.5个月（95%CI: 12.2-15.1），HR=0.88（95%CI: 0.73-1.05），*P*=0.158, 1。但是在PD-L1高表达或TC3/IC3的亚组中可见Atezolizumab联合化疗的OS优势，23.4个月（95%CI: 17.8-NE）*vs* 10.2个月（95%CI: 7.1-17.5），HR=0.48（95%CI: 0.29-0.81），但这一亚族样本量仅有91例，谨慎看待阳性结果。此外国内的PD-1抑制剂在晚期肺鳞癌的一线治疗中有积极探索，Orient-12研究对比了信迪利单抗联合吉西他滨/顺铂对比吉西他滨/顺铂一线治疗晚期肺鳞癌的Ⅲ期研究已完成入组，此外正在进行中的类似研究包括卡瑞利珠单抗联合紫杉醇/卡铂对比紫杉醇/卡铂一线治疗晚期肺鳞癌的Ⅲ期研究和特瑞普利单抗也在晚期NSCLC一线开展的联合标准化疗对比标准化疗的Ⅲ期研究，期待上述研究的结果为晚期鳞癌患者提供更多选择。综上，目前在晚期肺鳞癌一线治疗免疫联合化疗的模式中，Pembrolizumab联合卡铂/紫杉醇或白蛋白紫杉醇应是生存最佳的治疗方案。

免疫治疗中除了对PD-1及PD-L1抑制剂的研究，也有CTLA-4抑制剂联合化疗的探索。一项CTLA-4抑制剂Ipilimumab联合紫杉醇/卡铂对比紫杉醇/卡铂一线治疗晚期肺鳞癌的Ⅲ期研究^[50]^显示联合免疫治疗组的中位OS无统计学优势[13.4个月*vs* 12.4个月，HR=0.91（95%CI: 0.77-1.07），*P*=0.25]，且联合组TRAE发生率更高（3/4级TRAE发生率：51% *vs* 35%；任何级别TRAE发生率：33% *vs* 10%；导致治疗终止的TRAE发生率：28% *vs* 7%），联合组治疗相关死亡7例，对照组仅1例。CTLA-4抑制剂联合化疗未能获得生存获益。

双药免疫是另一个晚期一线联合治疗的方向。去年ESMO也报道了CheckMate 227研究中免疫联合免疫治疗一线对比标准化疗的研究结果^[51]^，在PD-L1≥1%的患者中，Nivolumab 3 mg/kg *q2w*联合Ipilimumab 1 mg/kg *q6w*对比标准化疗（腺癌：培美曲赛联合顺铂或卡铂；鳞癌：吉西他滨联合顺铂或卡铂）获得了生存获益，中位OS 17.1个月*vs* 14.9个月，HR=0.79（95%CI: 0.65-0.96），*P*=0.007，在PD-L1 < 1%的患者中Nivolumab联合Ipilimumab的生存仍较单纯化疗更优，中位OS 17.2个月*vs* 12.2个月，HR=0.62（95%CI: 0.48-0.78），所有随机患者中，免疫联合免疫对比化疗的生存优势明显，中位OS 17.1个月*vs* 13.9个月，HR=0.73（95%CI: 0.64-0.84），而在鳞癌亚组，免疫治疗的生存获益更为明显，中位OS 15.0个月*vs* 9.2个月（HR=0.62），从安全性来讲，Nivolumab联合Ipilimumab的治疗相关AE发生率略低于化疗组（77% *vs* 82%），免疫治疗不良反应以腹泻、皮疹、乏力为主，而化疗组仍以恶心、贫血、食欲降低为主。Nivolumab联合Ipilimumab未来也可能成为晚期肺鳞癌一线治疗的标准治疗。

在PD-1/PD-L1抑制剂、CTLA4抑制剂外的免疫治疗靶点也在不断探索中，IDO酶抑制剂Epacadostat分别联合Pembrolizumab和Atezolizumab的两项Ⅰ期研究均已完成^[52, 53]^，毒性可耐受，但目前尚无该药物在NSCLC的Ⅲ期研究开展。可见其他靶点的免疫治疗探索仍然充满挑战。

综上，晚期肺鳞癌的二线治疗中，Nivolumab、Pembrolizumab和Atezolizumab从疗效和安全性上均优于多西他赛，因此成为二线治疗的推荐方案。Pembrolizumab为PD-L1≥1%的晚期肺鳞癌一线患者提供更多的治疗选择，在晚期鳞癌一线最优的方案应该是Pembrolizumab联合卡铂/紫杉醇或白蛋白紫杉醇。Nivolumab联合Ipilimumab未来也可能成为晚期肺鳞癌的一线标准治疗方案。更多的免疫治疗药物仍在探索中。

## 5 展望

晚期肺鳞癌的治疗中，化疗仍是基石，针对特殊人群需综合评估年龄、PS评分、器官储备功能及治疗意愿等选择合适强度的治疗方案；鳞癌的靶向治疗仍在艰难的探索中，虽然FGFR TKI的探索屡屡受挫，但对于鳞癌常见突变的靶向治疗探索仍有重要意义和价值；由于作用机制及不良反应等原因，抗血管治疗对于晚期肺鳞癌的价值更多体现在后线治疗；仍在对肺鳞癌这样的突变复杂、高免疫原性肿瘤，免疫治疗似乎更有前景，如何利用生物标志物精准识别获益人群、如何能使免疫治疗在更广的人群中发挥更多作用、免疫治疗联合其他治疗（化疗、放疗、靶向治疗）的协同机制如何及是否具有可行性等，这些都需要在未来的研究中深入探索。
